# Critical Appraisal for Racial and Ethnic Equity in Clinical Prediction Models Extension: Development of a Critical Appraisal Tool Extension to Assess Racial and Ethnic Equity-Related Risk of Bias for Clinical Prediction Models

**DOI:** 10.1089/heq.2023.0035

**Published:** 2023-11-30

**Authors:** Shazia M. Siddique, Corinne V. Evans, Michael Harhay, Eric S. Johnson, Jaya Aysola, Gary E. Weissman, Nikhil K. Mull, Emilia Flores, Harald Schmidt, Kelley Tipton, Brian Leas, Jennifer S. Lin

**Affiliations:** ^1^ Division of Gastroenterology, Department of Medicine, University of Pennsylvania Perelman School of Medicine, Philadelphia, Pennsylvania, USA.; ^2^ Center for Evidence-Based Practice, University of Pennsylvania Health System, Philadelphia, Pennsylvania, USA.; ^3^ Kaiser Permanente Evidence-Based Practice Center, Kaiser Permanente Center for Health Research, Portland, Oregon, USA.; ^4^ Department of Biostatics, Epidemiology and Informatics, University of Pennsylvania, Philadelphia, Pennsylvania, USA.; ^5^ Penn Medicine Center for Health Equity Advancement, University of Pennsylvania Health System, Philadelphia, Pennsylvania, USA.; ^6^ Pulmonary, Allergy, and Critical Care Division, Department of Medicine, University of Pennsylvania Perelman School of Medicine, Philadelphia, Pennsylvania, USA.; ^7^ Department of Medical Ethics and Health Policy, University of Pennsylvania, Philadelphia, Pennsylvania, USA.; ^8^ ECRI, Plymouth Meeting, Pennsylvania, USA.

**Keywords:** health equity, risk of bias, critical appraisal, systematic review, guideline development

## Abstract

**Introduction:**

Despite mounting evidence that the inclusion of race and ethnicity in clinical prediction models may contribute to health disparities, existing critical appraisal tools do not directly address such equity considerations.

**Objective:**

This study developed a critical appraisal tool extension to assess algorithmic bias in clinical prediction models.

**Methods:**

A modified e-Delphi approach was utilized to develop and obtain expert consensus on a set of racial and ethnic equity-based signaling questions for appraisal of risk of bias in clinical prediction models. Through a series of virtual meetings, initial pilot application, and an online survey, individuals with expertise in clinical prediction model development, systematic review methodology, and health equity developed and refined this tool.

**Results:**

Consensus was reached for ten equity-based signaling questions, which led to the development of the Critical Appraisal for Racial and Ethnic Equity in Clinical Prediction Models (CARE-CPM) extension. This extension is intended for use along with existing critical appraisal tools for clinical prediction models.

**Conclusion:**

CARE-CPM provides a valuable risk-of-bias assessment tool extension for clinical prediction models to identify potential algorithmic bias and health equity concerns. Further research is needed to test usability, interrater reliability, and application to decision-makers.

## Introduction

Though it has long been argued that the use of race and ethnicity in medicine is problematic, only more recently, there have been widespread efforts to mitigate the use and consequences of race-based or race-informed medical decision-making.^1,2^ There are numerous problems with using race and ethnicity in medicine.^3^ Foremost is that its use perpetuates the incorrect and harmful notion that race is biologic. Racism, however, may have negative biological consequences via epigenetics.^4^ Further issues are that most racial and ethnic categories are overly broad and do not account for individuals that are multiracial or multiethnic background.^5^

A key area where scrutiny has emerged is the use of race and ethnicity in clinical prediction models. Specifically, there are many examples of clinical prediction models that may contribute to health and health care inequities by sustaining or exacerbating biases.^1,2^ A prime example is equations to calculate the estimated glomerular filtration rate (eGFR), including a race correction. This race correction erroneously resulted in higher estimates of kidney function in Black patients, which can delay specialist referrals and transplantation, contributing to inequities. Through efforts from a national task force, the equation was ultimately revised to remove race alongside early efforts to consider cystatin C biomarkers as an alternate to creatinine.^6^ However, for many decades, the eGFR algorithm was inherently biased, and this has shed light on the importance of re-examining other prediction models used in practice.^1^

On the other hand, scholars have identified examples where the removal of race and ethnicity from prediction models worsened algorithmic bias compared to a race-neutral model.^7^ These examples underscore need to directly assess algorithmic bias related to race and ethnicity to evaluate issues related to model design, data, and sampling that may disproportionately affect prediction model performance across racial and ethnic groups.

Critical appraisal tools allow systematic reviewers and other potential end users to objectively, transparently, and consistently assess and report on studies' risk of bias (RoB). However, existing tools, such as the Prediction model Risk of Bias Assessment Tool (PROBAST), do not specifically address risks of bias in the context of specific racial and ethnic groups and whether the application of a model to diverse populations may have health equity implications. Though efforts are underway to establish standards for using and reporting race and ethnicity in research broadly and in algorithms more specifically, many of these efforts are nascent, and they need to be integrated into existing tools for assessing RoB.^8–13^ In this study, we aimed to develop a critical appraisal tool extension to assess race and ethnicity related RoB for clinical prediction models.

## Methods

The Agency for Healthcare Research and Quality (AHRQ) commissioned the Evidence-Based Practice Center (EPC) program to develop methods to evaluate RoB in the development and validation of clinical prediction models that include race or ethnicity as a predictor or stratifying factor (i.e., are “race-aware”).^10^ The evaluation of RoB specific to the inclusion of race and ethnicity in clinical prediction models was intended to build upon the existing PROBAST^14^ tool and is conceptualized as an extension named the Critical Appraisal for Racial and Ethnic Equity in Clinical Prediction Models (CARE-CPM). The CARE-CPM was piloted and assessed using several prediction models in the primary care setting. To further refine this RoB extension, a modified e-Delphi process was utilized to determine consensus on equity-based signaling questions among a group of experts.

### Stage 1: scope, initial pilot, and definitions

The core team from the Kaiser Permanente EPC developed a framework from which to develop a racial and ethnic equity extension tool to assess algorithmic bias (CARE-CPM).^10^ The CARE-CPM was developed to maintain the four-domain structure of PROBAST, with signaling questions addressing potential risks of bias related to participants, predictors, outcome, and analysis. The extension questions were developed by first assessing which of the original PROBAST questions needed to be applied at the level of specific racial and ethnic groups as answers differing from assessment of the overall population may give rise to bias. Additionally, key concepts from foundational literature in algorithmic bias were added to the extension by phrasing as questions and applying consistent directionality to answer options.^3,9,13,15,16^ An initial set of questions was piloted on four prediction models to illustrate the feasibility and challenges in assessing the risk of algorithmic bias in published models, explore model limitations concerning race and ethnicity, and describe opportunities for further enhancements to directly address potential RoB as they relate to the inclusion of race and ethnicity.

To further refine the CARE-CPM extension, a larger steering group comprising 14 individuals among the AHRQ EPC teams at ECRI-Penn and Kaiser Permanente convened in a series of virtual meetings to discuss and provide feedback. Our steering committee has spent several years leading methods development to address racism, promote health equity, and evaluate evidence in specific populations for use in systematic reviews and clinical practice guidelines.^5,17–20^ This steering group included experts in health equity, clinical prediction models, evidence synthesis, guideline development, and an ethicist. For broader applicability, we agreed that the questions the questions would be relevant to any clinical prediction model, regardless of whether race and ethnicity were included as a predictor.

### Stage 2: online Delphi process

The Delphi technique is an established method to reach an expert consensus that has been used extensively to develop critical appraisal tools.^21,22^ It is characterized by two or more rounds of discussion and questionnaires with anonymity, controlled feedback, and statistical analysis of group response. For this study, we utilized a modified e-Delphi approach, which used virtual meetings and online surveys as the consensus-building model. A questionnaire was sent to a broader group of experts in clinical prediction model development, RoB assessment, and guideline development. We limited the administration of the survey to experts affiliated with US-based institutions, given the country's unique historical and current events that have shaped views on assessing racial and ethnic bias in health care.

Direct e-mails were sent to the expert panel containing a link to a questionnaire consisting of one overarching question on essential concepts to capture for the CARE-CPM extension and 11 questions related to proposed equity signaling questions within CARE-CPM. A 10-point numerical scale was used to capture the level of agreement. No identifying information was collected. An explanation of the rationale and considerations for each question were provided as informational text for each question. At the end of each question, feedback for open-ended comments and suggestions was requested in a text box but was not required to complete the survey. It was determined *a priori* that a mean agreement score of >7 out of 10 for each item would be the threshold to determine that a consensus agreement was reached. If agreement was reached, no further revision would be necessary. The core team of investigators at ECRI-Penn and Kaiser Permanente could incorporate open-ended feedback from survey responses to improve the clarity and usability of the CARE-CPM extension.

### Stage 3: refining the tool

Our steering group of 14 individuals incorporated survey feedback to further refine and clarify the CARE-CPM extension. Survey responses with a lower mean score, a higher standard deviation, or substantive open-ended feedback to clarify wording were prioritized for revision.

### Ethics statement

A presubmission inquiry was sent to the Institutional Review Board at the University of Pennsylvania, and it was determined that formal submission was not necessary as there was no collection of identifiable data from experts.

## Results

### Stage 1: scope, initial pilot, and definitions—results

The CARE-CPM extension was piloted with four clinical prediction models to assess feasibility, illustrate the challenges in assessing the risk of algorithmic bias, explore model limitations with respect to race and ethnicity, and describe opportunities for further refinements to directly address potential RoB as they relate to the inclusion of race and ethnicity. Here, we discuss the results of applying the CARE-CPM extension to one example, the Pooled Cohort Equations (PCE) for atherosclerotic cardiovascular disease.^14^

Several items could not be assessed by CARE-CPM when evaluating the PCE because no information was reported: the proportion of individuals in the development data set with missing data (for the overall population and by race and ethnicity); the potential for differential follow-up (for the overall population and by race and ethnicity); and exploration of model overfitting and optimism. Because of these reporting limitations, the initial PROBAST rating was “High RoB” and the rating remained “High RoB” with the addition of the CARE-CPM extension. As a result, the use of CARE-CPM did not significantly impact domain ratings for PROBAST. The lack of reporting may be because the PCE was developed before modern reporting guidelines for multivariable prediction models.^15^

Despite no changes in the RoB rating with use of the CARE-CPM, this tool identified issues that could contribute to algorithmic bias. Because the PCE was not developed with a competing risks model, and Black Americans suffer a higher age-specific all-cause mortality, the predicted 10-year probabilities of a cardiovascular event from the PCE's Cox model may be overestimated—an overestimation that would be worse than in White Americans. Further, smaller sample sizes (numbers of events for Black individuals) likely led to model overfitting in equations for this population. Additionally, the use of multiple imputation to handle missing data would be preferable for reducing selection bias; instead, the PCE excluded participants with missing predictors. If there are differences in the number of participants with missing data by race, a further selected and less representative sample would be used. While not required by PROBAST, the lack of confidence intervals for expected to observed events precludes firm conclusions about how calibration compares in Black and White individuals. Additionally, the lack of specific PCE for Hispanic, Asian, and Native populations creates critical questions about the people to whom the PCE is applicable. The CARE-CPM tool allowed for a more thorough delineation of racial and ethnic equity-related concerns.

### Stage 2: online Delphi process—results

The survey was electronically distributed to 21 individuals, and the response rate was 43% (*N*=9). We recruited individuals with overlapping knowledge of clinical prediction models, critical appraisal and systematic reviews, health equity, and guideline development. Respondents could identify as an expert in more than one area. Among the respondents, 7 (78%) self-identified as a systematic reviewer, 5 (56%) as an academic researcher, 3 (33%) as a clinician, 2 (22%) as a clinical prediction model researcher, 1 (11%) as a health equity expert, and 1 (11%) as a guideline developer. In addition to the 9 survey respondents, feedback from our steering committee of 14 individuals was incorporated to develop questions based on a total of 25 experts.

Survey participants agreed on the overarching concepts that should be addressed by equity-based signaling questions, as shown in Table 1.

**Table 1.** Survey Responses for Concepts Encompassing Racial and Ethnic Equity-Related Risk of Bias Assessment in Clinical Prediction Models

Concept	Mean score (SD)
Method of collection/reporting for race/ethnicity data	4.44 (0.88)
Representation/generalizability of data across racial/ethnic subgroups	4.56 (0.73)
Transparent rationale for the use of race/ethnicity	4.67 (0.50)
Differential missingness of data within racial/ethnic groups	4.33 (0.87)
Model performance in racial/ethnic groups	4.22 (0.83)

Respondents were asked to rate agreement with each concept on a scale of 1–5, in which 1=strongly disagree and 5=strongly agree.

SD, standard deviation.

Survey participants demonstrated overall consensus, with a mean score of >7 for every question. Mean agreement scores ranged from 7 to 9.25 (Table 2). Open-ended remarks were provided by several participants for suggestions to improve the wording of the questions.

**Table 2.** Delphi Responses with Agreement Rating for Each Racial and Ethnic Equity-Based Risk of Bias Assessment Question

Proposed CARE-CPM signaling question	Mean score (SD)
Were data on racial and ethnic groups gathered directly from participants, using consistent definitions or categories?	9.25 (1.0)
Was the racial and ethnic distribution of the population in the development data similar to the distribution in the target population?	7.88 (1.9)
Were racial and ethnic groups classified/categorized in a similar way in the development data and population to whom model is applied? (Validation studies only)	8.63 (1.3)
Was a transparent rationale provided for including race and ethnicity as a predictor variable or stratifying factor?	8.63 (1.5)
Did the model avoid using race and ethnicity as a proxy for a biological, social, or other risk factor that could be measured with more accuracy or fidelity? (Development studies only)	7.5 (2.9)
Was differential missingness of predictor data in racial and ethnic groups reported?	8.63 (1.2)
Was differential follow-up or ascertainment of the outcome in racial and ethnic groups reported?	9 (1.1)
Were proxy outcomes avoided as the predicted outcome, where the meaning of the proxy may differ in racial and ethnic groups (label choice bias)?	8.125 (1.9)
Were there sufficient outcomes in racial and ethnic groups to assess model performance separately in these groups? (Model validation studies)	8.5 (1.2)
Was differential life expectancy in racial and ethnic groups accounted for using competing risk methods?	7 (2.4)
Were relevant model performance measures evaluated appropriately in racial and ethnic groups? How does model performance (calibration, discrimination) compare among racial and ethnic groups?	8.63 (1.32)

Respondents were asked to rate agreement with each concept on a scale of 1–10, in which 1=strongly disagree and 10=strongly agree.

CARE-CPM, Critical Appraisal for Racial and Ethnic Equity in Clinical Prediction Models.

### Stage 3: refining the tool—results

Since consensus was reached on all questions, a second round of questionnaire distribution with the full group of experts was not needed. However, in an effort to incorporate feedback provided in the survey responses, a final e-Delphi round among experts from the EPC teams at ECRI-Penn and Kaiser Permanente was conducted to further revise the questions. As a response to feedback, one item was consolidated into another question given interrelated nature, the wording of four items was changed, and the rationale text was modified for six items. The final set of 10 race and ethnicity equity-based questions for the CARE-CPM Extension is shown in Table 3.

**Table 3.** Revised Racial and Ethnic Equity-Based Signaling Questions for Critical Appraisal for Racial and Ethnic Equity in Clinical Prediction Models Extension

PROBAST domain	Added signaling questions	Rationale and considerations
1. Participants	1.3a. Were data on racial and ethnic groups gathered using consistent definitions or categories with adequate response options?	The classification of race and ethnicity is complex, and there are no best practices for collecting these data.^3^ For example, self-reported race, race as categorized by others, and family racial history each reflect distinct meanings of race. In the absence of best practices, at minimum, the collection of racial and ethnic data should use consistent categories and definitions. Considerations include the availability of a category for multiracial/multiethnic individuals and whether heterogeneity within groups is addressed (e.g., the identification of Black or Indigenous heritage within the broader Hispanic category, country of origin, or immigration status). Ideally, the collection of race and ethnicity should adhere to evidence-based REAL (Race, Ethnicity, and Language) standards with the ability to select multiple categories.^11^
	1.3b. Was the racial and ethnic distribution of the population in the development data similar to the distribution in the target population?	Representation bias occurs when estimates from one population are inappropriately extrapolated to other populations.^12^ Underrepresentation of racial and ethnic groups may contribute to differential predictive performance. The population distribution from development data could be compared to recent Census estimates at the national, state, or local level to assess representativeness in the target population.
	1.4. Were racial and ethnic groups classified/categorized in a similar way in the development data and population to whom model is applied? (Validation studies only)	Similar to 1.3a, but in this case, were the categories consistent between development and validation datasets, or was there further opportunity for misclassification?
2. Predictors	2.4a. Was a transparent rationale provided for including race and ethnicity as a predictor?	In the absence of consensus or clear criteria for the inclusion of race and ethnicity in clinical prediction models,^9^ at minimum, is the rationale for inclusion of race and ethnicity transparent? For example, do authors state that race and ethnicity was included to improve calibration because of known differences in incidence? While prediction modelers are not asked to justify the inclusion of other variables, it is appropriate in the case of race and ethnicity because of concerns regarding potential misuse. Race should not be included in models with a causal aim because the notion of racial and ethnic groups as genetically distinct has been invalidated.^23^ For causal inference, the social construct of race could be decomposed into causal elements such as more direct measures of racism,^24^ health care access, socioeconomic status, or biologic differences due to chronic stressors.
	2.5. Was differential missingness of predictor data in racial and ethnic groups reported?	Missing data may be associated with contact with the health care system which in turn reflects care patterns and structural racism. Exclusion of participants with missing data provides a selective sample of data for model development.^14^ Bias may be further exacerbated in the context of differential missingness among groups. The more missing data there is in a population, the more selected and less representative the development data become when used in a complete case analysis where participants with missing data are excluded. Multiple imputation is the preferred method for handling missing data so that data from all participants can be included; this is further addressed in PROBAST item 4.4.^14^
3. Outcome	3.4a. Was differential follow-up or ascertainment of the outcome in racial and ethnic groups reported?	Differential ascertainment of outcomes among groups may lead to systematic over or underprediction. Similarly, differential loss to follow up among groups may result in differences in censoring among groups. Censoring can bias predicted risks because of overrepresentation of those experiencing the outcome.^14^
	3.7. Were proxy outcomes avoided as the predicted outcome, where the proxy may be subject to encoded bias (label choice bias)?	Label choice bias is a mismatch between the ideal target the algorithm should be predicting and a biased proxy variable the algorithm is actually predicting.^15^ Proxy outcomes may reflect encoded bias where some racial and ethnic groups have experienced less access to a service and the use of this measure as a predicted outcome could reinforce inequities. For example, health care cost may reflect access rather than true health needs.^16^ Similarly, revascularization as an event of interest may reflect practice patterns favoring intervention in specific groups.^25^
4. Analysis	4.1a. Were there sufficient outcomes occurring in specific racial and ethnic groups to assess model performance separately in these groups? (Model validation studies)	The effective sample size in prediction models is the number of outcome events. This question asks whether the events per variable are adequate when assessed separately in racial and ethnic groups. Investigators have suggested 10–20 events per variable in development studies, but the actual calculation can be more complex.^26^ In validation studies, at least 100 events is recommended.^27^
	4.6a. Were competing risk methods used in the prediction model?	Overestimation and bias can occur in prediction models not accounting for prominent competing risks, such as all-cause mortality in elderly populations.^26^ Standard predictions using Cox regression models can overestimate absolute risk because individuals with a competing event (e.g., all-cause death) are censored and treated as if the predicted outcome could occur in the future.^28^ The potential for bias is further exacerbated in the context of well documented differential life expectancy among racial and ethnic groups. A similar question was recently added in the QUAPAS tool.^29^
	4.7a. Were relevant model performance measures evaluated appropriately in racial and ethnic groups? How does model performance (calibration, discrimination) compare among racial and ethnic groups?	Both calibration and discrimination should be assessed and reported separately by racial and ethnic group, allowing for comparison among groups. Reported measures of calibration should be meaningful, such as a calibration plots or expected to observed ratios, which allow for a quantification of the direction and magnitude of any miscalibration. Additional guidance available in Moons et al.^26^ Because model performance is quantified using a number of different measures, assessments of algorithmic bias may differ for each measure with respect to population characteristics (i.e., case mix) and varying condition prevalence or incidence. For example, discrimination can be higher in samples with greater population variability.^30^ While differences in discrimination measures are expected in different groups, accurate models will always have good calibration across groups.

PROBAST, Prediction Model Risk of Bias Assessment Tool.

## Discussion

In this modified e-Delphi process, we developed a new set of equity-based signaling questions for RoB assessment of clinical prediction models, termed the Critical Appraisal for Racial and Ethnic Equity in Clinical Prediction Models (CARE-CPM) extension. The goal for the CARE-CPM extension is to serve as an addendum to existing critical appraisal tools, such as the PROBAST tool, which provide important methodologic assessment specific to clinical prediction models but does not address specific equity concerns as they relate to race and ethnicity.

As with PROBAST and other related RoB tools to assess clinical prediction models, users of the tool should have both subject and methodologic expertise. The application of the CARE-CPM extension may change the individual domain ratings from the application of PROBAST alone and thus change the overall RoB assessment for a clinical prediction model. However, as shown in the pilot application of these questions before the e-Delphi process, there was no change in overall RoB assessment with the addition of these questions, mostly driven by the high RoB rating as determined by the original PROBAST signaling questions alone. In this scenario, utilizing the CARE-CPM extension allowed reviewers to better articulate racial/ethnic equity concerns with each model. This, in turn, allows guideline developers, researchers, and policymakers to more comprehensively and directly determine the potential consequences of utilizing clinical prediction models to inform decision-making.

Features of the CARE-CPM extension can be incorporated into standards for model developers to use as guidance to minimize racial and ethnic bias. Such reporting standards provide a useful opportunity to transparently address issues relating to race and ethnicity, and also allow for the quantification of algorithmic bias needed to better inform clinical recommendations. However, it is important to note that both critical appraisal tools and reporting standards alone are not sufficient to account for potential biases in prediction models within the evidence pipeline. For greatest impact, learnings from the critical appraisal process can be fed back into upstream model development or revision, or even further upstream to data collection processes to facilitate improvements to models. If model revision is not possible, detailed knowledge of model limitations can be used to design more equitable implementation strategies.

There are limitations of this work. First, we included US-based participants only. This was done intentionally given current heightened awareness about systemic racism and health/health care inequities in the United States. In the United States, there has been recent greater awareness of how systemic racism influences the development of clinical prediction models and subsequent application in a population (e.g., eGFR), beyond issues purely related to model calibration and performance. Second, the survey was distributed to a relatively select and small group of individuals. This was done intentionally as we aimed to identify experts with overlapping knowledge of prediction models, RoB assessment, and health equity. Finally, the response rate was modest (43%), though, this is similar to prior survey-based studies of health care workers and methodologists.^31^

Beyond the limitations of this individual study, there is a need to gain broader consensus from systematic review and guideline developers internationally regarding handling the inclusion of race and ethnicity in clinical prediction models. Specifically, there are ongoing debates as to whether there are justified scenarios to include race and ethnicity in clinical prediction models, and whether improved model calibration is sufficient to warrant the inclusion of these variables that could be misused. Several examples of clinical prediction models have shown potential harm to marginalized populations, for example, equations to estimate GFR which have directed resources away from Black individuals.^32^ Opportunities for race-aware clinical prediction models to direct resources toward communities experiencing health inequities may be possible.^7,33,34^

The CARE-CPM extension warrants further study, including an assessment of internal rating consistency within a selected evidence review and ideally an evaluation of how RoB ratings correspond to quantitative assessment of the direction and magnitude of algorithmic bias of a model. Additionally, further modification of these questions to apply to other study designs will allow for them to be adapted and extended to other existing critical appraisal tools as considerations of equity should be explicit in all research designs. Specifically, the CARE-CPM extension will need to be applied to a broader evidence base to test for internal validity (e.g., consistency among systematic reviewers) before the tool can be broadly applied.

Large studies have demonstrated that the vast majority of published clinical prediction models have a high RoB as assessed by PROBAST.^35^ This high RoB is present even without the application of the CARE-CPM. This suggests that the extension of critical appraisal to include racial and health equity considerations will not change the ultimate RoB assessment in most cases as the CARE-CPM will render already strict criteria even stricter. Despite no changes in ultimate RoB “grade,” by undergoing a consistent and transparent process of considering RoB specific to racial and ethnic groups, a user will have the tools to identify and articulate model limitations that could result in health equity concerns if the model is implemented. Such information can be used to inform model redesign or implementation practices to address equity flaws. We believe this is an important step in shaping future guideline recommendations that stem from prediction models so that an equity lens is applied when assessing the strength of evidence.

Thus, with increasing awareness of the potential inequitable implications of clinical prediction models, there was an unmet need to incorporate race and ethnicity equity considerations into RoB assessment. This modified e-Delphi process reached a consensus for a set of equity-based race and ethnicity signaling questions for clinical prediction models. Further application of the CARE-CPM extension and pragmatic adaptations of the tool are needed to guide the utilization of the equity-based signaling questions.

## Abbreviations Used

AHRQ: Agency for Healthcare Research and Quality
CARE-CPM: Critical Appraisal for Racial and Ethnic Equity in Clinical Prediction Models
eGFR: estimated glomerular filtration rate
EPC: Evidence-Based Practice Center
PCE: Pooled Cohort Equations
PROBAST: Prediction Model Risk of Bias Assessment Tool
RoB: risk of bias
SD: standard deviation
